# Age Deficits in Associative Memory Are Not Alleviated by Multisensory Paradigms

**DOI:** 10.1093/geronb/gbae063

**Published:** 2024-04-25

**Authors:** Stephen P Badham, Christopher Atkin, Jemaine E Stacey, Helen Henshaw, Harriet A Allen, Katherine L Roberts

**Affiliations:** NTU Psychology, Nottingham Trent University, Nottingham, UK; NTU Psychology, Nottingham Trent University, Nottingham, UK; NTU Psychology, Nottingham Trent University, Nottingham, UK; Hearing Sciences, Mental Health, and Clinical Neurosciences, School of Medicine, University of Nottingham, Nottingham, UK; National Institute for Health and Care Research (NIHR), Nottingham Biomedical Research Centre, Nottingham, UK; School of Psychology, University of Nottingham, Nottingham, UK; NTU Psychology, Nottingham Trent University, Nottingham, UK; (Psychological Sciences Section)

**Keywords:** Associative deficit hypothesis, Episodic memory, Multisensory stimuli, Paired associates, Sensory deficits

## Abstract

**Objectives:**

Age deficits in memory are widespread, this affects individuals at a personal level, and investigating memory has been a key focus in cognitive aging research. Age deficits occur in memory for an episode, where information from the environment is integrated through the senses into an episodic event via associative memory. Associating items in memory has been shown to be particularly difficult for older adults but can often be alleviated by providing support from the external environment. The current investigation explored the potential for increased sensory input (multimodal stimuli) to alleviate age deficits in associative memory. Here, we present compelling evidence, supported by Bayesian analysis, for a null age-by-modality interaction.

**Methods:**

Across three preregistered studies, young and older adults (*n* = 860) completed associative memory tasks either in single modalities or in multimodal formats. Study 1 used either visual text (unimodal) or video introductions (multimodal) to test memory for name-face associations. Studies 2 and 3 tested memory for paired associates. Study 2 used unimodal visual presentation or cross-modal visual-auditory word pairs in a cued recall paradigm. Study 3 presented word pairs as visual only, auditory only, or audiovisual and tested memory separately for items (individual words) or associations (word pairings).

**Results:**

Typical age deficits in associative memory emerged, but these were not alleviated by multimodal presentation.

**Discussion:**

The lack of multimodal support for associative memory indicates that perceptual manipulations are less effective than other forms of environmental support at alleviating age deficits in associative memory.

One of the most salient and established age-related changes in cognition is a reduction in memory performance, which occurs in healthy aging (e.g., Luo & Craik, 2008) but also to a greater extent with onset of dementia (e.g., Arvanitakis et al., 2019). Correspondingly, memory performance represents a key concern for older individuals and fear of memory loss is shown to predict lower quality of life in the older population (Farnina et al., 2022). Memory studies feature heavily in cognitive aging research due to the prevalence of memory loss and the impact it can have on individuals’ lives. Crucially, however, the profile of age-related change in memory performance is not uniform and the comparison of stable and declining memory abilities offers the potential to uncover mechanisms underpinning age-related cognitive change. Some memory abilities such as semantic and implicit memory show minimal age-related change, whilst others such as working memory and explicit memory tasks appear more susceptible to age-related decline (see Park & Festini, 2017, for a review). Establishing methods of alleviating or modulating age differences in memory can therefore offer practical methods for improving older adults’ quality of life at the same time as developing theory about processes that are more susceptible or less susceptible to age-related decline. The current study aimed to establish if experiencing stimuli through multiple sensory modalities (auditory plus visual) could alleviate age deficits in associative memory.

Within the domain of explicit memory, associative memory has been shown to be particularly difficult for older adults (Old & Naveh-Benjamin, 2008). Older adults show larger memory deficits relative to young adults for associations between items (e.g., a name and a face) compared to age deficits shown on memory for individual items (e.g., recognizing a face as seen before). Naveh-Benjamin (2000) presented an associative deficit hypothesis of aging, and argued that across a variety of paradigms, the age deficit in memory could be explained at least partly by the degree to which a memory task required associations between individual units of information. Since this conceptualization, much research has been undertaken to establish mechanisms linked to the associative deficit hypothesis. It has been shown with paired associates that semantic links between word pairs (e.g., flashlight-candle) disproportionately support older adults’ ability (relative to young adults) to remember associations between individual words (e.g., Badham et al., 2012). This indicates that prior knowledge and experience could alleviate age deficits in memory and have the potential to support older adults’ memory performance (Badham et al., 2016). Research has also shown that activating stereotypes of older adults having poor memory led to greater age-related associative deficits than a no-threat condition (Brubaker & Naveh-Benjamin, 2018). Other methods of reducing the age-related associative deficit also have clear potential for improving memory in general; Anderson et al. (2018) achieved this through a training task and Naveh-Benjamin et al. (2007) achieved this through the encouraging the use of encoding and retrieval strategies. Together, these examples suggest that associative memory performance is malleable and with effective support can be improved in the older population. This is particularly promising if one adopts the view that associative memory is an essential component of episodic memory, responsible for binding the components of an episode (sound, vision, context) into a cohesive episodic trace (see Shing et al., 2010, for a review).

The factors shown to alleviate age deficits in associative memory above are congruent with theory surrounding environmental support. In his early work, Craik (1986) highlighted how age deficits in memory were smaller when information and cues could be utilized from the environment to support memory. For example, smaller age deficits in memory are found in recognition, where stimuli are represented at retrieval (high support) compared to free recall, where the participant must engage with self-initiated processing (low support) to retrieve individual items (e.g., Craik & McDowd, 1987; Rhodes et al., 2019). This framework has become one of the most prominent accounts used to explain patterns of age deficits (e.g., Luo & Craik, 2008; Park & Festini, 2017) and featured heavily in Naveh-Benjamin’s (2000) conceptualization of the associative deficit hypothesis. This is because associative memory is thought to require effortful, strategic processing more aligned with free recall paradigms, whilst item memory is thought to be more heavily supported by automatic processes aligned with recognition (Naveh-Benjamin, 2000). The modulation of age-related associative memory deficits in paradigms above indicates that environmental support can encourage effective self-initiated processing in older adults through a variety of methods. Namely, support via (i) familiarity with stimuli (Badham et al., 2012), (ii) training/practice (Anderson et al., 2018), and (iii) encouragement of mnemonic strategy use (Naveh-Benjamin et al., 2007).

One mechanism of processing support that is yet to be applied to age-related associative memory deficits is the presentation of stimuli in multiple modalities. The meta-analysis by Old and Naveh-Benjamin (2008) indicated that memory involving associating information to its modality of presentation showed minimal associative deficits, so paradigms appealing to modality support may be particularly effective at utilizing intact processing abilities of older adults. Most notably, recent reviews have highlighted how older adults may disproportionately (relative to young adults) make use of multisensory processing in cognitive tasks (Brooks et al., 2018; Dieuleveult et al., 2017; Freiherr et al., 2013). This is partially thought to be because as sensory ability declines due to aging (Roberts & Allen, 2016), older adults seek to compensate by drawing upon more sensory input (De Dieuleveult et al., 2017).

There are longstanding observations that a positive relationship exists between sensory ability and cognitive performance in older adults (see Li & Lindenberger, 2002; Pichora-Fuller et al., 2017; Roberts & Allen, 2016; Schneider & Pichora-Fuller, 2000, for reviews), and deficits in memory have been induced in young adults by raising sensory demands with noisy/degraded stimuli (e.g., Murphy et al., 2000), including memory deficits in associative memory (Naveh-Benjamin & Kilb, 2014). It has been hypothesized that as sensory perception declines, cognitive resources related to perceiving stimuli may detract from mnemonic processing such as encoding and consolidation (see cognitive shift hypothesis; Schneider & Pichora-Fuller, 2000). Recent research has shown that older adults can utilize multisensory information to improve item memory to the same extent as young adults (Atkin et al., 2023). Therefore, theory predicts that utilizing multisensory stimuli may also provide environmental support to older adults’ associative memory processes, possibly to an even greater extent than young adults (cf. De Dieuleveult et al., 2017), such that multisensory stimuli might alleviate age deficits in associative memory. However, despite these predictions derived from current understanding, in the remainder of this article, we present three distinct, theoretically motivated paradigms that demonstrate multimodal stimuli have minimal effect when used to alleviate associative memory deficits in older adults.

## Study 1: Name-Face Associations

This study focused on the naturalistic associative memory task of associating a name to a face. Difficulty remembering a person’s name is one of the leading memory complaints among older adults and failure can lead to social embarrassment, resulting in a source of stress for older individuals (Biss et al., 2018). This paradigm has received much attention in the associative memory literature including adjusting the nature of recognition lures (Fine et al., 2018), adjusting the amount of exposure to stimuli (Biss et al., 2018), and relating name-face associative ability to subjective memory complaints and dementia risk factors (Horn et al., 2018). In the current study, we manipulated the presentation modality of names alongside images of faces. In a unimodal, visual-only condition names were presented via text, and in a multimodal condition, names were presented by a video introduction. It was hypothesized that the multisensory condition would support older participants’ associative memory more than young participants’ associative memory.

## Method

### Design

**Figure 1.**
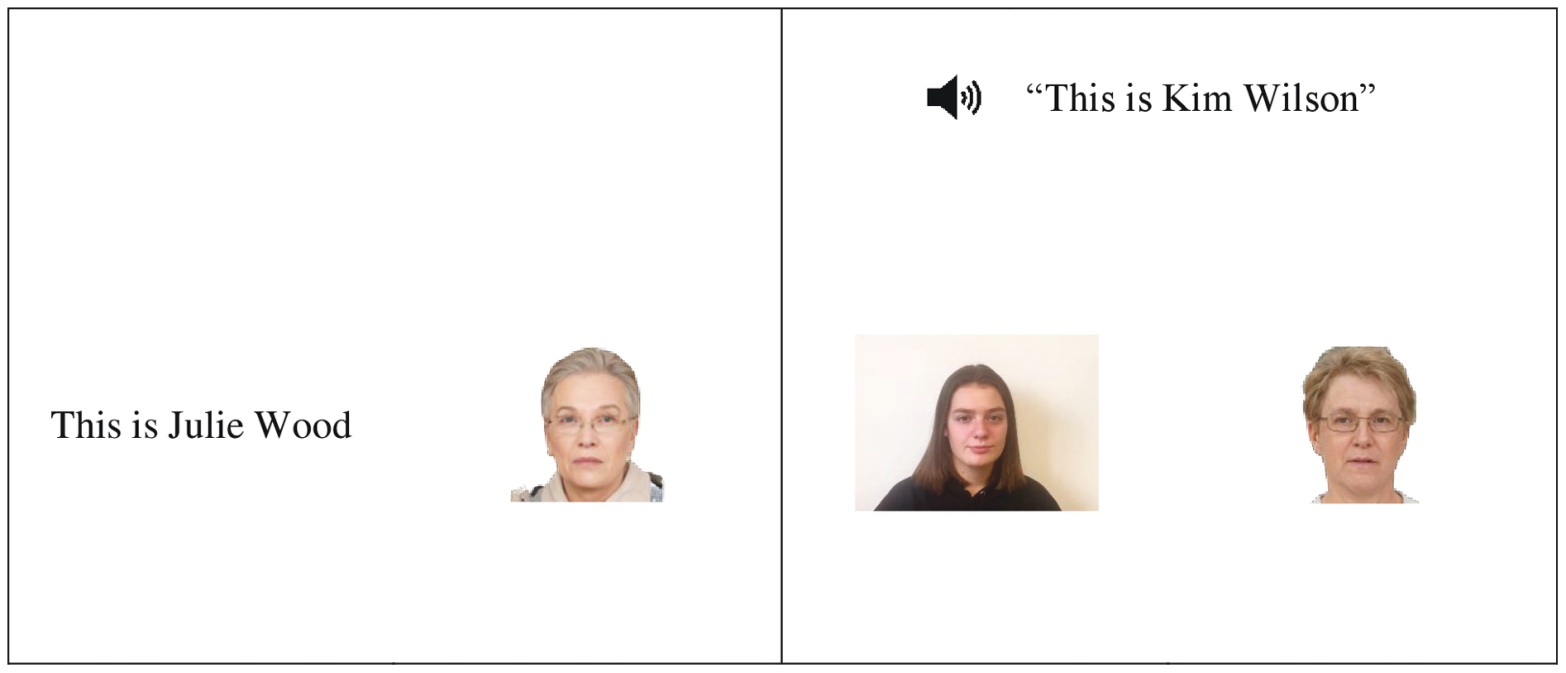
Example unisensory (left) and multisensory (right) encoding trial from Study 1. Face images here are for illustrative purposes to protect the integrity of the FACES database. The video screenshot is from the experiment.

Young and older adults were tested on a list of name-face associations, where names were presented either in visual only or audiovisual modality between participants (Figure 1).

### Participants

Sixty young and 66 older participants took part in the experiment online and were recruited on Prolific and were compensated £1.80. Table 1 shows the demographic characteristics of the samples for all studies in the article. Sample sizes were derived from previous research, as outlined in the preregistration linked in the *Data Availability* section. Throughout this article, all research was given a favorable ethics opinion by Nottingham Trent University’s Research Ethics Committee.

**Table 1.** Participant Background Measures for Studies 1–3

Measure	Study 1		Study 2		Study 3	
	Young	Older	Young	Older	Young	Older
*N* (*N* male, *N* female)^a^	60 (20, 38)	66 (25, 41)	72 (21, 49)	72 (36, 36)	298 (148, 144)	292 (147, 145)
Age, Mean (*SD*)	25.35 (3.14)	65.91 (3.90)	25.19 (3.13)	70.25 (4.25)	25.34 (3.17)	73.71 (3.87)
Age, range	19–30	60–76	18–31	65–80	18–30	68–94
SSQ12,^b^ Mean (*SD*)	7.20 (1.44)	7.59 (1.41)	6.86 (1.46)	7.47 (1.63)^*^	7.66 (1.25)	7.61 (1.39)
ADVS,^c^ Mean (*SD*)	4.75 (0.22)	4.63 (0.24)^**^	4.72 (0.29)	4.65 (0.26)	4.68 (0.38)	4.40 (0.56)^**^

^a^Total *N* also includes gender not specified as male or female.

^b^Subjective hearing: Speech, Spatial, and Qualities of Hearing scale (SSQ12).

^c^Subjective vision: Activities of Daily Vision Scale (ADVS).

^*^ *p* < .05.

^**^ *p* < .001 for age difference.

### Materials

Subjective hearing was measured by the Speech, Spatial, and Qualities of Hearing scale (SSQ12, Noble et al., 2013). A higher score on this 12-item questionnaire indicates better auditory functioning.

Subjective vision was measured by the Activities of Daily Vision Scale (ADVS, Mangione et al., 1992). The scale measures visual functioning in different situations and consists of 21-multiple choice questions, which are rated on a scale from 1 to 5. A higher score on this questionnaire indicates better visual functioning.

Each participant saw 12 faces (6 female and 6 male) taken from Ebner et al. (2010) with perceived ages between 43 and 50 (to avoid own-age bias in young and older adults). First and last names were generated on Name Generator (name-generator.org.uk). Participants were randomly shown one of two versions of the experiment, which had different names and faces (a total of 24 names and 24 faces were used across conditions).

Faces were either introduced with text stating “This is [Name]” displayed to the left of the image (visual only unimodal condition) or with a short video of a young woman against a plain white background stating “This is [Name]” with audio to allow speechreading (see Figure 1). Video was used instead of audio to approximately equate the amount of visual information on the screen across conditions.

### Procedure

Stimuli were displayed in the software Gorilla (https://Gorilla.sc). Demographics, ADVS, and SSQ12 were completed followed by either the unisensory or multisensory task. In the multisensory condition, a sound check was provided where the participant could set an appropriate volume. In both sensory conditions, each face was then displayed for 5 s with a 1-s interstimulus interval (ISI) during encoding. Between encoding and retrieval there was a 30-s period of backwards counting in threes. Following this, the retrieval period commenced. Participants were presented with a name and a face in the same modality/modalities as encoding and were required to respond old/new via a button press. Half of the trials had intact name-face pairs (old) and half were recombined by recombining names and faces from the encoding phase into novel pairs (new). Each retrieval trial stayed on screen until a response was made. There was a 1-s ISI between each retrieval trial, old/new trials were randomized.

## Results

Throughout this article, analyses were completed in IBM’s SPSS Statistics version 28, and inclusive Bayes factors were computed using JASP (JASP Team 2022; version 0.16.1). Inclusive Bayes factors (BF_Inclusive_) provide the odds of the alternative hypothesis over the null hypothesis (e.g., BF_Inclusive_ = 2.5 means that the alternative hypothesis is 2.5 times more likely than the null hypothesis). All analyses are included in Supplementary Materials.

Signal detection theory was used to compute *d*ʹ as a measure of accuracy and ln beta as a measure of response bias using Stanislaw and Todorov (1999). A 2 (Age Group: Young/Older) × 2 (Presentation Modality: Visual text/video introduction) between-participants ANOVA was conducted on the *d*ʹ data (see Table 2 for means). There was no main effect of age group, *F*(1, 122) = 0.74, *p* = .391, η^2^ = 0.006, BF_Inclusive_ = 0.214. In contrast to our hypothesis, the video introduction condition proved to be more difficult than the visual text condition with frequentist analysis (although this was less conclusive with Bayes analysis), *F*(1, 122) = 4.61, *p* = .034, η^2^ = 0.036, BF_Inclusive_ = 1.183. Crucially, there was no evidence that the older group disproportionately benefitted from multisensory stimuli as there was no interaction between age group and presentation modality, *F*(1, 122) = 0.63, *p* = .803, η^2^ = 0.001, BF_Inclusive_ = 0.143, with the BF_Inclusive_ value indicating moderate (Quintana & Williams, 2018) evidence in favor of the null hypothesis. The same pattern was found using hit rates minus false alarm rates (see Supplementary Materials).

**Table 2.** Mean (*SD*) *d*ʹ Sensitivity and Response Bias Values for Study 1

Age group	Presentation modality	*dʹ*	ln beta
Young	Visual text (unimodal)	1.30 (1.10)	−0.28 (0.42)
	Video introduction (multimodal)	0.97 (0.97)	−0.13 (0.44)
Older	Visual text	1.19 (0.85)	−0.30 (0.35)
	Video introduction	0.78 (0.95)	0.03 (0.30)

The same ANOVA structure was used to analyze ln beta response bias (see Table 2, for means). There was no main effect of age group, *F*(1, 122) = 1.12, *p* = .293, η^2^ = 0.009, BF_Inclusive_ = 0.347. Responses were more biased toward responding old/seen before in the visual text condition compared to the video introduction condition, which was relatively unbiased, *F*(1, 122) = 13.13, *p* < .001, η^2^ = 0.097, BF_Inclusive_ = 61.12. There was no interaction between the two factors, *F*(1, 122) = 1.87, *p* = .174, η^2^ = 0.015, BF_Inclusive_ = 0.56.

Finally, there was just one correlation between SSQ12 and accuracy, but only for young adults, *r*(58) = 0.43, *p* < .001 (uncorrected for multiple comparisons, see Supplementary Materials). The Supplementary Materials also include serial position analyses computed as part of the review process that were not preregistered. There were no effects of serial position.

### Summary of Study 1

Using a multisensory spoken video introduction instead of a text only visual introduction reduced young and older adults’ memory performance, with both age groups showing similar effects due to the modality manipulation, as indicated by moderate evidence for a null interaction between age group and presentation modality. Literature suggested older adults would find the multimodal condition more useful (De Dieuleveult et al., 2017) but this is difficult to interpret here as the multisensory condition was potentially more difficult, counter to our expectations. However, in studies where the multisensory information is distracting/negative, older adults can show larger multisensory effects (e.g., Guerreiro & Van Gerven, 2011) but this was also not the case with the current data.

It was also unusual to see no significant age deficit in memory performance in this task, which contrasted much literature (e.g., Biss et al., 2018; Fine et al., 2018; Horn et al., 2018). However, the numerical pattern was consistent with prior results, and the current sample of older adults was slightly young (*M* = 66, 60–76). One factor that could have influenced this was the use of online testing, which may self-select more able older adults (Badham et al., 2022) this was accounted for in Study 2, as well as the mean age of the older sample.

## Study 2: Cross-Modal Binding

As Study 1 showed only numerical age deficits, Study 2 aimed to utilize a paired associates memory paradigm more popularly used to evoke age differences in associative memory. This would therefore have more potential for older adults’ memory to be improved by a multisensory manipulation. This cued recall paradigm also was more likely to increase age deficits in memory compared to the recognition-based paradigm in Study 1 (e.g., Craik & McDowd, 1987; Rhodes et al., 2019). Also, to maximize the potential for multisensory stimuli to aid memory, in this study, the multimodal condition involved cross-modal binding, where one word within a pair was presented visually and the other word was presented auditorily; prior research has shown that mixed modality presentation can aid verbal memory, potentially by appealing to different underlying phonological and visuospatial storage systems (Frick, 1984). Finally, this study was also conducted both online and in the laboratory to test for potential sampling biases in online aging research.

## Method

### Design

The cued recall paradigm replicated Badham et al. (2012) in the unimodal condition—pairs of words were shown visually at encoding. In the multimodal condition, stimuli were presented cross-modally, with the cue word of each pair presented visually and the target word of each pair presented auditorily. The overall design was age group (young/older) by experiment location (between participants; online/in laboratory) by modality (within participants; unimodal/cross-modal).

### Participants

Seventy-two young and 72 older participants took part in the experiment. Online participants were recruited on Prolific and were compensated £2, laboratory-tested older adults were compensated £10 (other research was completed in the same laboratory session: spatial memory and a choice reaction task) and older adults were recruited from a standing panel of volunteers, whilst young adults were recruited from the university campus. Table 1 shows the demographic characteristics of the samples for all studies in the article. In addition, young adults (*M* = −11.23, *SD* = 3.17) were significantly better than older adults (*M* = −6.69, *SD* = 3.48) in an objective measure of hearing (CRM, Bianco et al., 2021), *t*(142) = 8.19, *p* < .001. Sample sizes were derived from previous research as outlined in the preregistration linked in the *Data Availability* section.

### Materials

The memory stimuli were acquired from the appendix of Badham et al. (2012), which consists of a set of 45 paired unrelated words (e.g., carrot—money). Spoken versions of the words (male voice) were generated on Wideo (https://wideo.co). Memory lists were 17 word pairs with the middle 15 used to test for cued recall, excluding the first and last pairs to avoid primacy and recency effects (Badham et al., 2012).

Subjective hearing and vision were measured using the SSQ12 and ADVS as described in Study 1. Additionally, participants completed an objective hearing test developed for online testing that involved perception of speech in noise (an adapted CRM task; Bianco et al., 2021).

### Procedure

Participants completed the hearing and vision measures and then completed two memory tests, one visual-only test and one cross-modal visual-auditory test. Stimuli were displayed in the software Gorilla (https://Gorilla.sc). Memory test order was counterbalanced between participants. Participants were instructed to study the word pairs with full knowledge that the memory test would involve cuing with the left word of each pair and generating the right (or spoken) word of each word pair at test. Encoding was the only aspect of the task that differed between conditions. At encoding, the left word of each pair was displayed for 500 ms and stayed on screen as the right word appeared next to it either visually for 5,000 ms (visual only condition) or in conjunction with it as an auditory spoken word of approximately 500-ms duration over a static screen continuing to display just the left word for 5,000 ms total (multimodal condition). There was a 500-ms ISI between word pairs. Between encoding and retrieval, there was a 30-s period of backwards counting in threes. Retrieval involved being shown the left word of each pair on screen with the participant typing in the word that they remembered being presented alongside it. Extra methodological detail on timings and randomization can be found in the preregistration.

## Results

A 2 (Age Group: Young/Older) × 2 (Experiment Location; Online/Laboratory; between participants) × 2 (Modality: unimodal/cross modal; within participants) repeated measures ANOVA was conducted on the proportion of correctly recalled paired associates (see Table 3, for means). Young adults showed better memory than did older adults, *F*(1, 140) = 16.76, *p* < .001, η^2^ = 0.107, BF_Inclusive_ = 129.1, there was no main effect of experiment location, *F*(1, 140) = 0.867, *p* = .353, η^2^ = 0.107, BF_Inclusive_ = 0.362, and no main effect of modality, *F*(1, 140) = 3.66, *p* = .058, η^2^ = 0.025, BF_Inclusive_ = 0.387. None of the interactions was significant: Age Group × Experiment Location, *F*(1, 140) = 2.00, *p* = .159, η^2^ = 0.014, BF_Inclusive_ = 0.678, Age Group × Modality, *F*(1, 140) = 1.52, *p* = .219, η^2^ = 0.011, BF_Inclusive_ = 0.330, Modality × Experiment Location, *F*(1, 140) = 0.644, *p* = .424, η^2^ = 0.005, BF_Inclusive_ = 0.127, Age Group × Experiment Location × Modality, *F*(1, 140) = 0.381, *p* = .538, η^2^ = 0.003, BF_Inclusive_ = 0.041. Finally, there were no correlations linking self-rated perception measures, or speech in noise ability to memory performance after controlling for multiple comparisons (see Supplementary Materials).

**Table 3.** Mean (*SD*) Proportion Accuracy Values for Study 2

Age group	Experiment location	Unimodal	Multimodal
Young	Online	0.57 (0.36)	0.56 (0.36)
	In laboratory	0.59 (0.31)	0.59 (0.34)
Older	Online	0.45 (0.26)	0.43 (0.23)
	In laboratory	0.36 (0.29)	0.29 (0.24)

### Summary of Study 2

The only effect in this study was that of age, with typical age-related associative deficits demonstrated in the data (Badham et al., 2012; Naveh-Benjamin, 2000). It was good to see no main effects or interactions involving testing location (online vs in laboratory), given the uptake in online data collection (Sassenberg & Ditrich, 2019). Crucially, there were no effects of modality, with both age groups similarly unaffected by the modality manipulation, and with borderline moderate/anecdotal evidence for the null interaction between age and modality. These results indicate that older adults do not demonstrate any difficulty in multimodal integration, consistent with some evidence (see Jones & Noppeney, 2021, for a review) but they are inconsistent with arguments suggesting increased multisensory use in older adults (De Dieuleveult et al., 2017).

## Study 3: Age-Related Associative Deficit Paradigm With a Multimodal Manipulation

Studies 1 and 2 showed that young and older adults appear to be relatively similar across manipulations of presentation modality in terms of associative memory, even when an age deficit is present (Study 2). In the earlier studies, the multimodal condition required memory for information in both modalities to successfully complete each trial. In Study 3, this was not the case, we used a popular paradigm from the seminal paper of Naveh-Benjamin (2000) involving memory for word pairs with tests of both individual words and their pairings. The unimodal conditions were either visual only or auditory only and the multimodal condition was audiovisual. Therefore, the multimodal condition allowed visual and auditory information to support each other during encoding and retrieval. Additionally, following thorough pilot work, we calculated statistical power for this specific paradigm, allowing confident assessment of the null hypotheses.

## Method

Extra methodological detail is provided in Supplementary Materials (https://osf.io/7x86n/).

### Design

Unrelated word pairs were studied, followed by separate recognition tests for item memory (individual words) and associative memory (word pairings). Young and older adults completed the item and associative tests (within participants). Participants completed the experiment in one of three modalities: visual only, auditory only, or audiovisual (between participants, with modality matched at encoding and retrieval).

### Participants

The experiment comprised 298 young and 292 older participants. Participants were recruited on Prolific and were paid as £2.50 as compensation. Table 1 shows the demographic characteristics of the samples. We performed a power analysis using the WebPower package in R (Zhang & Yuan, 2018) for an interaction effect with the ANOVA structure outlined above in the design section. We specified a medium effect size based on a marginal interaction in pilot data (registered at https://aspredicted.org/blind.php?x=1QL_52Z) with 120 independent participants. Our results indicated that a sample size of approximately 449 would be needed to achieve a statistical power of 0.8, and the current study had a power of 0.91.

### Materials

Unrelated word pairs were taken from Badham et al. (2012) as in Study 2. Spoken versions of the words (male voice) were generated on Wideo (https://wideo.co). At encoding, participants studied 24 word pairs. At retrieval, for the item test, 8 of these word pairs were broken up to form 16 old (seen before) individual word stimuli. These were combined with a further 16 unseen words to form a 32-trial old/new item recognition memory test. For the associative memory test, the remaining 16 pairs from the encoded word pairs that were not used in the item memory test were used as stimuli. Intact word pairs were formed from 8 of the 16 pairs. Recombined word pairs were formed from the remaining 8 word pairs by taking a word from one pair and combining it with a word from another pair. Together, this formed a 16-trial intact/recombined associative recognition memory test.

The SSQ12 and ADVS self-rated measures of hearing and vision were also collected as described in Study 1.

### Procedure

Participants completed either a visual-only version of the experiment, an auditory-only version of the experiment or an audiovisual version of the experiment. Stimuli were displayed in the software Gorilla (https://Gorilla.sc). For all versions, participants were asked to remember the words and their pairings for a later memory test (explicit memory). The word pairs were displayed visually at encoding at a rate of 5 s per pair with a 450-ms ISI. The auditory-only and audiovisual versions played the sound files automatically at 0 ms and 1,000 ms into each trial. Between encoding and retrieval there was a 30-s period of backwards counting in threes. The modality of presentation was the same during encoding and retrieval. Memory was tested via old/new responses on the keyboard.

## Results

**Figure 2.**
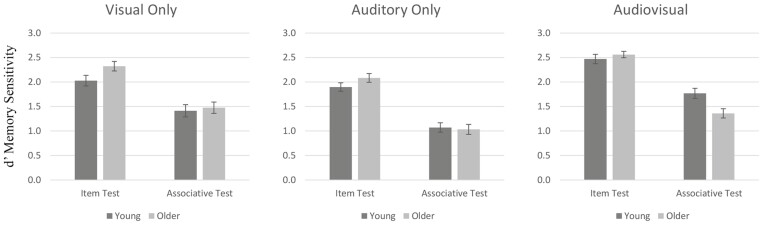
Memory performance (*d*ʹ) in Study 3. Error bars are ± 1SE.

A 2 (Age Group: Young/Older) × 3 (Modality; Visual Only/Auditory Only/Audiovisual; between participants) × 2 (Test Type: Item/Associative; within participants) repeated measures ANOVA was conducted on *d*ʹ sensitivity (see Figure 2, for Means). There was no main effect of age, *F*(2, 584) = 0.203, *p* = .652, η^2^ = .000, BF_Inclusive_ = 9.38, a main effect of modality was driven by auditory-only being more difficult than the visual-only and multimodal conditions, *F*(2, 584) = 18.31, *p* < .001, η^2^ = 0.059, BF_Inclusive_ = 3.47 × 10^5^, and the associative memory test was harder than the item memory test, *F*(1, 584) = 468.35, *p* < .001, η^2^ = 0.445, BF_Inclusive_ = 2.77 × 10^13^. The only interaction with age was the age-related associative deficit, which showed a greater drop in performance from item to associative memory in older adults compared to young adults, *F*(1, 584) = 15.63, *p* < .001, η^2^ = 0.026, BF_Inclusive_ = 48.96. There was also an interaction between test type and modality, such that the visual only condition differed from the audiovisual condition for the item test but not the associative test, *F*(1, 584) = 3.10, *p* = .046, η^2^ = 0.010, BF_Inclusive_ = 0.805. There was no interaction between age and modality, *F*(2, 584) = 2.01, *p* = .131, η^2^ = 0.007, BF_Inclusive_ = 0.651. Crucially, the triple interaction was nonsignificant, *F*(2, 584) = 1.27, *p* = .282, η^2^ = 0.004, BF_Inclusive_ = 0.133, with the Bayes factor indicating the null hypothesis was 7.5× more likely than the alternative hypothesis. This meant that there was moderate evidence (Quintana & Williams, 2018) that the age-related associative deficit was not alleviated by multisensory stimuli.

The remaining analyses can be found in Supplementary Materials as well as serial position analyses that were not preregistered. There were no effects of serial position. In brief, the only interaction with age on response bias (ln beta) consistent across frequentist and Bayesian analysis was such that young and older adults had similar, minimal bias on the associative memory test but older adults were more biased towards “no” responses for the item memory test, *F*(2, 584) = 7.77, *p* = .005, η^2^ = 0.013, BF_Inclusive_ = 3.66. There were also no correlations between demographics/self-rated sensory measures and memory performance.

### Summary of Study 3

This study successfully replicated the age-related associative deficit, but the deficit was not modulated by the sensory modality of memory stimuli. Bayesian evaluation of the null hypothesis indicated that there was evidence against theory that would predict multisensory stimuli could alleviate age deficits (De Dieuleveult et al., 2017), instead the data showed similar utilization of multisensory stimuli in both age groups (cf. Jones & Noppeney, 2021).

## General Discussion

Across three studies, it was consistently found that young and older adults responded similarly to manipulations of modality in associative memory tasks. Study 1 used a naturalistic name-face association task, older adults’ performance was as good as young adults’ performance, and multimodal video introductions of each name were *harder* to memorize than visual text introductions of each name, in contrast to expectations. Study 2 utilized a paired associates cued recall task, an age-related associative deficit was present, but memory was similar for word pairs encoded visually and for cross-modal binding with one visual and one auditory word in each pair. Study 2 additionally showed similar performance for online versus in-laboratory testing. Study 3 also utilized a paired associates paradigm but utilized recognition tests of both item (individual words) and associative (word pairings) memory, with stimuli presented either visual only, auditory only, or multimodal. Here, the multimodal condition therefore contained redundant information. Auditory only presentation was more difficult to remember than visual-only and multimodal presentation. In Study 3, a large sample (*n* = 590) led to the null hypothesis being 7.5 times more likely than the alternative hypothesis, indicating moderate evidence for no modulation of age differences in associative deficits by modality.

The general pattern in the current data is consistent with other aging research that shows no age by modality interactions in the domain of free recall (Atkin et al., 2023; Constantinidou & Baker, 2002) and with conclusions by Jones and Noppeney (2021), who argued multisensory processing is intact in older adults. It was expected that multimodal stimuli would facilitate perceptual processing that would translate to a reduction in the age-related associative deficit (cf. De Dieuleveult et al., 2017). However, it seems that adjusting processing demands does not influence the associative deficit. For example, studies increasing processing demands via divided attention manipulations show similar divided attention costs for item and associative memory (Craik et al., 2010). Looking back at the studies alleviating the age-related associative deficit by environmental support, it appears as though most are operationalized through strategic support such as scaffolding retrieval with prior knowledge (Badham et al., 2016), training/practice (Anderson et al., 2018) and direct strategy instruction (Naveh-Benjamin et al., 2007). Shing et al. (2010) presented an account of episodic memory that dissociated a strategic component from an associative component across the lifespan and they argued that when environmental support facilitates associative memory it does so in the strategic domain. Therefore, we conclude that multimodal perceptual manipulations may offer no strategic benefit to older adults’ associative memory. This is despite evidence of multimodal support of memory in general compared to auditory-only processing (current Study 3; Atkin et al., 2023; Constantinidou & Baker, 2002).

### Limitations and Future Directions

A potential limitation in the current study is that no relations were found between self-reported sensory measures and memory performance (or objective hearing and memory in Study 2). It has been argued earlier that multisensory enhancement may stem from sensory deficits (e.g., De Dieuleveult et al., 2017), although De Dieuleveult et al. (2017) also summarized some studies showing older adults’ multisensory enhancement in the absence of unisensory deficits. Furthermore, in a separate study from our laboratory, we also found no multisensory enhancement of memory among older hearing aid users (Stacey et al., under review).

Another potential limitation of the current study is the use of online testing. This limited the use of objective sensory measures and there may be systematic differences in the way that young and older adults utilize IT equipment (cf. Mariano et al., 2022). However, Study 2 showed similar effects in online- and laboratory-tested participants. Finally, Study 3 utilized a within-participants interaction paradigm that compared age differences in item memory to age differences in associative memory, which self-controlled for variance in IT use and still showed no modality modulation of age-related associative deficits.

The rich multimodal nature of episodic memory in the real world (cf. Shing et al., 2010) may be more heavily influenced by the amount of available multimodal information than in experimental work. For example, age differences exist in lipreading (Feld & Sommers, 2009), which may affect multimodal manipulations involving speechreading in real-life contexts. Furthermore, very little has been done with multimodal stimuli in modalities other than sound and vision: One study showed odor could disproportionately support older adults’ memory (relative to young adults) for source information (Gilbert et al., 2006), whilst another study showed enhanced autobiographical memory when prompted by odor, to a similar extent in both young and older adults (Maylor et al., 2002).

Based on the current findings, the next step could be to utilize modality to encourage strategy utilization. Frick (1984) used lists with mixed modality to aid in chunking in a working memory task and to minimize interference between items. Additionally, Badham and Maylor (2013) showed that modality could heavily aid memory in an isolation effect paradigm, although the effect was similar for young and older adults. Alternatively, other factors demonstrating boundary conditions on the effect of environmental support on associative memory could help further dissociate strategic from associative processing in episodic memory.

## Data Availability

All data sets and analytical output are available in the Supplementary Materials. All of the research was preregistered and the preregistrations are as follows: *Study 1:* https://aspredicted.org/QQ4_CKZ *Study 2:* Online experiment: https://osf.io/ge4rz; in laboratory plus online experiment: https://osf.io/dkxyj *Study 3:* https://aspredicted.org/J3D_LX4
